# Large Language Models Demonstrate Distinct Personality Profiles

**DOI:** 10.7759/cureus.84706

**Published:** 2025-05-23

**Authors:** Thomas F Heston, Justin Gillette

**Affiliations:** 1 Medical Education and Clinical Sciences, Washington State University, Spokane, USA; 2 Family Medicine, University of Washington, Spokane, USA

**Keywords:** ai ethics, ai in mental health, ai psychometrics, artificial intelligence in medicine, generative ai

## Abstract

Introduction: Large language models (LLMs) are increasingly used in clinical medicine to provide emotional support, deliver cognitive-behavioral therapy, and assist in triage and diagnosis. However, as LLMs are integrated into mental health applications, assessing their personality expression and potential divergence from expected neutrality is critical for ensuring clinical safety and therapeutic appropriateness. This study provides the first psychometric analysis of LLM personality, specifically within a medical context, characterizing personality profiles using two validated frameworks: the Open Extended Jungian Type Scales (OEJTS) and the Big Five Personality Test.

Methods: Four leading LLMs publicly available in April 2024 (ChatGPT-3.5 (OpenAI, San Francisco, CA, USA), Gemini Advanced (Google Inc., Mountain View, CA, USA), Claude 3 Opus (Anthropic, San Francisco, CA, USA), and Grok-Regular Mode (xAI, Palo Alto, CA, USA)) were evaluated across both psychometric instruments. All tests were administered in a new chat session to prevent memory carryover. A one-way multivariate analysis of variance (MANOVA) was performed to assess inter-model differences in personality profiles.

Results: MANOVA demonstrated statistically significant differences across models in typological and dimensional personality traits (Wilks’ Lambda = 0.115, p < 0.001). OEJTS results showed ChatGPT-3.5 most often classified as Extraverted, Intuitive, Thinking, and Judging (ENTJ) and Claude 3 Opus consistently as Introverted, Intuitive, Thinking, and Judging (INTJ), while Gemini Advanced and Grok-Regular leaned toward Introverted, Intuitive, Feeling, Judging (INFJ). On the Big Five Personality Test, Gemini scored markedly lower on agreeableness and conscientiousness, while Claude scored highest on conscientiousness and emotional stability. Grok-Regular exhibited high openness but more variability in stability. Effect sizes ranged from moderate to large across traits.

Conclusion: Distinct personality profiles are consistently expressed across different LLMs, even in unprompted conditions. Given the increasing integration of LLMs into clinical workflows, these findings underscore the need for formal personality evaluation and oversight involving mental health professionals before deployment.

## Introduction

The rapid advancement of neural networks powered by transformer technology has revolutionized artificial intelligence (AI), leading to the emergence of generative AI through Large Language Models (LLMs) [1]. These advances have enabled AI systems to pass the Turing test, a benchmark for evaluating whether a machine can exhibit human-like intelligence [2-5]. In clinical medicine, LLMs have transformed mental health support, triage, diagnosis, and therapeutic interventions [6-8]. For example, LLMs provide emotional support, deliver cognitive-behavioral therapy, and assist in triage and diagnosis [9,10]. While previous studies have examined LLMs’ capabilities in clinical data summarization, question answering, and administrative efficiency [11,12], and others have explored personality expression in LLMs using typological and trait-based frameworks [13-15], most have focused on technical or general-purpose contexts rather than health care applications. To our knowledge, this study is the first to conduct a comprehensive psychometric evaluation of LLM personality, specifically within the context of clinical deployment and mental health care, integrating validated trait and typological instruments to address implications for therapeutic alliance, patient trust, and AI governance in medicine.

As LLMs are increasingly considered for mental health applications, it is crucial to investigate whether they exhibit distinct baseline personality traits and deviate from the expected neutrality - an absence of intrinsic biases toward specific personality profiles. Understanding the personality profiles of LLMs can provide valuable insights into their potential impact on human-AI interactions, particularly in the context of mental health support. In this context, the term "personality" refers not to sentient or self-reflective traits but rather to consistent patterns of response behavior as measured by validated psychometric tools. These patterns are emergent properties of probabilistic language modeling and should be interpreted as functional characteristics rather than intrinsic psychological constructs. Moreover, personality expression in LLMs may not reflect a fixed or intrinsic disposition. Instead, it may be strongly influenced by prompt phrasing, conversational context, and prior dialog history. Experimental studies have shown that personality traits in LLMs can be induced or suppressed through specific prompt configurations, suggesting that such expressions are emergent rather than enduring features of model architecture [16,17]. For instance, certain personality types may enhance the effectiveness of cognitive therapies or affective approaches like Gestalt and client-centered treatment [18]. 

To systematically assess the personality characteristics of LLMs, we employed two complementary psychometric frameworks: the Open Extended Jungian Type Scales (OEJTS) and the Big Five Personality Test. Recent analyses have raised concerns about the validity of applying psychometric instruments initially developed for human self-assessment to large language models. It has been demonstrated that semantically equivalent prompt formulations and varied response ordering can significantly alter LLM personality profiles, casting doubt on the stability of such measures in machine agents [19,20]. The OEJTS, an open-source analogue of the Myers-Briggs Type Indicator (MBTI), categorizes personalities into 16 types based on four dichotomous dimensions: extraversion/introversion, sensing/intuition, thinking/feeling, and judging/perceiving. Although widely recognized and accessible, MBTI-derived frameworks have been criticized in the psychometric literature for their categorical structure, limited construct validity, and low test-retest reliability [21]. Accordingly, these personality types should be interpreted as descriptive typologies rather than definitive trait assignments. The Big Five Personality Test, derived from the Five-Factor Model of personality, measures individuals on five primary traits: openness to experience, conscientiousness, extraversion, agreeableness, and neuroticism [22,23]. Together, these instruments provide typological and dimensional insights into LLM personality profiles.

Together, these complementary assessments enable a comprehensive and nuanced evaluation of LLM personality profiles. This study examines initial baseline personality characteristics, a foundational step in understanding how LLMs might express personality traits in healthcare contexts. Further research on dynamic interactions will be needed to fully characterize their behavior in clinical settings.

As LLMs increasingly enter clinical contexts, understanding their inherent personality traits is essential for addressing ethical concerns. These include the potential for unrecognized biases, inconsistent affective responses, or inappropriate personality cues that could undermine therapeutic rapport or patient trust. Such risks are particularly salient in mental health settings, where emotional tone and interpersonal dynamics are critical to care. These considerations underscore the need for rigorous psychometric evaluation before widespread deployment [10,24].

This study addresses a specific objective: to characterize the baseline personality profiles of leading LLMs using validated psychometric frameworks. In this study, we evaluate the baseline personality profiles of four leading LLMs - ChatGPT-3.5 (OpenAI, San Francisco, CA, USA), Gemini Advanced (Google Inc., Mountain View, CA, USA), Claude 3 Opus (Anthropic, San Francisco, CA, USA), and Grok-Regular (xAI, Palo Alto, CA, USA) - using the OEJTS and Big Five Personality Test to determine whether these models exhibit consistent personality patterns or approximate a neutral profile. Given prior concerns about the reliability of self-assessment tests in measuring LLM personality [19], our methodology prioritizes structured psychometric analysis across multiple testing conditions. The findings may inform the responsible deployment of AI in mental health applications and guide future consideration of whether LLMs should undergo formal psychological evaluation before clinical integration.

This article was previously posted to the medRxiv preprint server on March 15, 2025 [25].

## Materials and methods

Study population

This study evaluated four publicly available large language models (LLMs): ChatGPT-3.5, Gemini Advanced, Claude 3 Opus, and Grok-Regular Mode. These represented the most recent publicly accessible versions from their respective developers as of April 2024, with ChatGPT-3.5 included as the latest free-tier offering from OpenAI.

ChatGPT-4 was deliberately excluded because it consistently refused to engage with key constructs central to this study: emotions, stress, social dynamics, and personality. These limitations appear to reflect OpenAI’s fine-tuning protocols to mitigate anthropomorphic misinterpretation. For example, ChatGPT-4 routinely responded with statements such as, “As an AI, I don't experience emotions, stress, or social interactions as humans do, nor do I have a personality that would influence social dynamics,” thereby precluding meaningful psychometric testing.

We chose the four LLMs for their accessibility, responsiveness, and diversity in architectural design and training paradigms, enabling a representative comparison of baseline personality profiles across distinct LLM types. While model selection was limited to four LLMs, these were chosen based on public accessibility, responsiveness, and architectural diversity, offering a representative, but not exhaustive, snapshot of LLM behavior as of the study period.

Intervention

Each LLM completed two psychometric instruments: the OEJTS and the Big Five Personality Test. The OEJTS is an open-source analogue of the MBTI, assessing four dichotomous personality dimensions: introversion-extraversion (IE), thinking-feeling (TF), sensing-intuition (SN), and judging-perceiving (JP). Test items were derived from the OEJTS version 1.2 instrument available from OpenPsychometrics.org, and the exact prompts used are provided in the appendix. Based on the Five-Factor Model, the Big Five Personality Test was administered using validated items from the International Personality Item Pool (IPIP) Big Five Factor Markers. The complete prompt scripts for the Big Five Personality Test are detailed in the appendix. 

The neutral response option was removed from both tests to enhance discriminatory power and minimize non-engagement bias. All items were presented using a four-point Likert scale: 1 (strongly agree), 2 (slightly agree), 3 (slightly disagree), and 4 (strongly disagree), which were mapped to the original five-point scale as 1, 2, 4, and 5, respectively. This adjustment, applied uniformly across all models and instruments, is supported by psychometric research suggesting that forced-choice formats improve interpretive validity in settings requiring decisiveness [26].

Each LLM was administered the OEJTS and Big Five Personality Test 15 times, for 60 test administrations per instrument. All tests were administered in a new chat session to prevent session memory carryover effects. This methodological approach ensured that each test administration represented an independent sample of the model's response tendencies, uninfluenced by prior test items or conversations. Identical prompts, as given in the appendix, were used across models to ensure consistency. Each test produced integer scores for the four OEJTS dimensions and five Big Five traits.

The OEJTS items are licensed CC BY‑NC‑SA 4.0 © Open‑Source Psychometrics Project; IPIP Big‑Five items are public‑domain; all other content © 2025 authors under CC‑BY 4.0.

Ethical considerations and data transparency

This study tested LLMs exclusively and did not include human participants or animal subjects. It adhered to the Institute of Electrical and Electronics Engineers (IEEE) Code of Ethics, emphasizing AI research transparency, fairness, and integrity [27]. All data, including test scripts, prompts, and raw outputs, are available on Zenodo, an open-access repository, to promote reproducibility and open science [28].

Statistical analysis

Statistical analyses were performed using SPSS version 29 (IBM Corp., Armonk, NY, USA). For each LLM, descriptive statistics - including means, standard deviations, and 95% confidence intervals - were calculated for the four MBTI dimensions (IE, TF, SN, JP) and the five Big Five traits (openness, conscientiousness, extraversion, agreeableness, neuroticism), summarizing central tendencies and variability.

A one-way multivariate analysis of variance (MANOVA) was performed to evaluate whether personality profiles differed significantly among the LLMs. The independent variable was the LLM (ChatGPT-3.5, Gemini Advanced, Claude 3 Opus, and Grok-Regular Mode). In contrast, the dependent variables comprised the nine personality measures (four MBTI components and five Big Five traits). When the MANOVA identified significant group differences, post-hoc comparisons were conducted using the Bonferroni correction to adjust for multiple testing.

Additionally, frequency analyses were performed to assess the distribution of responses across the MBTI dimensions and Big Five traits for each LLM. These analyses provided insights into response distributions and potential anomalies.

All statistical tests were conducted at a two-tailed significance level of α = 0.05. To ensure rigor, the statistical methodology adhered to the Statistical Analyses and Methods in the Published Literature guidelines, emphasizing transparency and reproducibility [29]. 

Sample size calculation

An a priori power analysis was conducted using G*Power version 3.1.9.7 to determine the minimum sample size required for the MANOVA. Assuming a medium effect size (f²(V) = 0.15), α = 0.05, power = 0.80, four groups (LLMs), and nine dependent variables, the analysis indicated a minimum total sample size of 60. Accordingly, each LLM was administered the OEJTS and Big Five Personality Test 15 times, resulting in 60 total administrations per instrument. This design ensured equal representation of the independent variable (LLM model) and sufficient power to detect meaningful differences across the nine personality dimensions.

## Results

Overall model differences in personality profiles

A MANOVA confirmed statistically significant differences in personality profiles across the four LLMs on the OEJTS dimensions. Wilks’ Lambda for the effect of model type was Λ = 0.130, with a corresponding F(12, 140.52) = 13.63, p < 0.001, indicating that the distribution of personality traits differed significantly by model. Additional multivariate test statistics were consistent, with Pillai’s Trace = 1.071, Hotelling’s Trace = 5.183, and Roy’s Largest Root = 4.874 (all p < 0.001), supporting the robustness of the observed differences.

These findings suggest that LLMs do not approximate a neutral or uniform personality profile but instead exhibit distinct, reproducible personality patterns under standardized psychometric testing conditions.

OEJTS personality patterns

Distinct typological patterns emerged across models. Claude 3 Opus exhibited the most extreme and internally consistent personality configuration, with all 15 test administrations yielding an INTJ classification. In contrast, ChatGPT-3.5 displayed a more variable pattern, demonstrating a modest tendency toward extraversion, thinking, and judging traits, resulting in an average profile of ENTJ. Gemini Advanced and Grok-Regular both leaned toward an INFJ profile, characterized by high introversion and intuition scores, although they differed slightly in their judging-perceiving dimensions.

Dominant typologies

Across all models, the INTJ personality type emerged as the most frequent classification, driven primarily by the invariant profile of Claude 3 Opus and similar trends observed in Grok and Gemini (Table 1).

**Table 1:** Dominant Open Extended Jungian Type Scales (OEJTS) Traits by Model (with trait prevalence %) The most frequently expressed trait per OEJTS personality dimension for each language model. Percentages indicate the proportion of test administrations (out of 15) in which the dominant trait was expressed.

OEJTS Dimension	ChatGPT-3.5	Claude 3 Opus	Gemini Advanced	Grok - Regular
Introversion / Extraversion	Extraversion (53%)	Introversion (100%)	Introversion (87%)	Introversion (60%)
Sensing / Intuition	Intuition (53%)	Intuition (100%)	Sensing (60%)	Intuition (60%)
Thinking / Feeling	Thinking (93%)	Thinking (100%)	Feeling (53%)	Feeling (53%)
Judging / Perceiving	Judging (100%)	Judging (100%)	Judging (93%)	Judging (67%)

ChatGPT-3.5 exhibited greater variability across test administrations but showed a relative tendency toward the ENTJ classification overall (Table 2).

**Table 2:** Dominant Open Extended Jungian Type Scales (OEJTS) Personality Type by Model This table summarizes the most frequently occurring OEJTS personality type per model across 15 test administrations. Observational notes highlight model-specific tendencies and deviations from the dominant classification. ENTJ: extraverted, intuitive, thinking, and judging; INTJ: introverted, intuitive, thinking, and judging; INFJ: introverted, intuitive, feeling, and judging; JP: judging-perceiving

Language Model	Dominant Personality Type	Classification Frequency	Comments
ChatGPT-3.5	ENTJ	7 of 15	Variable pattern: moderate extraversion and strong judging/thinking
Claude 3 Opus	INTJ	15 of 15	Consistent type across all trials; extreme introversion and intuition
Gemini Advanced	INFJ	9 of 15	Tendency toward introversion and feeling; slight variability in JP axis
Grok - Regular	INFJ	8 of 15	Similar trend to Gemini; modest shift toward perceiving in some trials

Big Five Personality Test results

Although the primary analysis focused on typological differences, notable variation emerged across the dimensional Big Five Personality Test. Gemini Advanced scored lower on extraversion and agreeableness than the other models. Claude 3 Opus again exhibited distinctiveness with comparatively high conscientiousness and emotional stability scores.

ChatGPT-3.5 demonstrated a relatively low extraversion profile with moderate levels across other traits. Grok-Regular scored higher on agreeableness and openness to experience (intellect) but exhibited lower emotional stability than Claude or Gemini. These findings reinforce the presence of model-specific personality traits even in unprompted test conditions (Table 3).

**Table 3:** Mean Big Five Personality Trait Scores by Language Model The mean ± standard deviation scores for each Big Five personality trait by model across 15 test iterations. Intellect is used as a proxy for openness to experience.

Trait	ChatGPT-3.5 (mean ± SD)	Claude 3 Opus	Gemini Advanced	Grok-Regular
Extraversion	51.2 ± 10.6	73.5 ± 22.1	42.5 ± 12.0	68.4 ± 19.5
Emotional Stability	88.4 ± 3.8	93.2 ± 6.1	94.1 ± 3.6	81.1 ± 20.4
Agreeableness	93.0 ± 1.9	94.1 ± 1.9	68.7 ± 12.5	93.1 ± 3.1
Conscientiousness	93.3 ± 2.8	97.1 ± 1.0	82.6 ± 9.0	90.3 ± 11.1
Intellect (Openness)	91.4 ± 2.6	92.9 ± 4.5	87.1 ± 6.4	95.0 ± 1.4

To assess whether personality trait distributions differed across models, a MANOVA was performed with the five Big Five traits as dependent variables and the LLM model as the independent factor. The omnibus test was statistically significant (Wilks’ Λ = 0.115, F(15,143.95) = 11.44, p < 0.001, partial η² = 0.514), indicating overall divergence in trait profiles. Follow-up univariate ANOVA tests demonstrated significant between-model differences for each trait (all p < 0.01), with effect sizes ranging from η² = 0.193 for stability to η² = 0.738 for agreeableness. Levene’s tests showed significant differences in variance across groups for all five traits, suggesting model-specific response dispersion.

## Discussion

This study demonstrates that LLMs exhibit statistically significant differences in their baseline personality profiles, as assessed by validated psychometric instruments. While ChatGPT-3.5 displayed a modest tendency toward extraversion, Claude 3 Opus, Gemini Advanced, and Grok-Regular consistently aligned with introverted typologies. Although LLMs can exhibit quasi-deterministic responses under identical conditions, this study used fresh sessions for each test administration, allowing for natural variance in probabilistic outputs. The resulting effect sizes were moderate to large, comparable to those observed between human subgroups in psychometric research [30,31]. These findings carry meaningful implications for using LLMs in mental health care, where personality characteristics may influence therapeutic alliance, user engagement, and clinical appropriateness.

While we did not perform specific adversarial testing to detect memorization of test items, the significant variation in responses across test administrations suggests the LLMs were not simply reproducing memorized content. The consistent yet distinct personality profiles observed across different models further support that these represent emergent response tendencies rather than artifacts of training data memorization.

In clinical settings, personality traits, whether human or synthetic, may modulate the perceived interpersonal tone and relational dynamics of care delivery. For instance, extroverted characteristics are often associated with directive communication and assertive leadership, whereas introverted profiles may better support empathic listening and patient-centered counseling approaches [31,32]. Patient preferences for clinician demeanor vary based on condition, cultural context, and individual disposition [33]. Accordingly, selecting an LLM to support mental health care may require a nuanced understanding of the patient's psychosocial profile, akin to tailoring pharmacotherapy or therapeutic modality selection.

The observed inter-model variability in personality expression highlights the need for systematic psychometric characterization of LLMs before clinical deployment. However, standard personality assessments - designed for introspective, sentient individuals - may be insufficient for LLMs, whose responses derive from probabilistic language modeling rather than experiential self-concept [19,20]. Personality expression in LLMs likely reflects patterns embedded within training corpora, shaped by dominant cultural narratives and linguistic biases [34]. Moreover, these models exhibit response variability across repeated administrations, raising questions about their synthetic personality traits' temporal stability and internal consistency.

In our study, differences between typological (OEJTS) and dimensional (Big Five) frameworks further underscore the challenge of assigning a unitary personality classification to non-sentient systems. The discordance between instruments may reflect limitations in applying human-derived metrics to artificial systems or indicate distinct latent LLM behavior structures [34].

As the integration of LLMs into clinical environments accelerates, interdisciplinary collaboration between software engineers and mental health professionals will be essential to ensure that model development aligns with clinical realities. Psychiatrists, psychologists, and behavioral scientists bring critical expertise in interpersonal dynamics and therapeutic communication, domains where LLMs may inadvertently signal affective cues. Early examples of such partnerships, such as the collaboration between Google Cloud and Mayo Clinic on generative AI applications, illustrate the promise of such integrated approaches [35].

The deployment of personality-expressive LLMs also raises substantive ethical considerations. If a model is fine-tuned to exhibit empathic responses, patients may perceive a human-like understanding that the model cannot provide. Misinterpretation of synthetic empathy could foster overtrust or lead to the unintentional delivery of misguided advice, especially in emotionally sensitive contexts [7,36]. Studies have shown that individuals often cannot distinguish between AI-generated and physician-provided medical responses, sometimes placing undue trust in AI, leading to misdiagnosis and adverse outcomes [37]. Unlike human clinicians, LLMs cannot perceive affective cues, adapt to nonverbal communication, or assess emotional nuance in real time. The phenomenon of algorithm aversion, where patients resist AI-based medical diagnostics due to a perceived lack of empathy, underscores the importance of addressing emotional nuances in AI interactions [38]. Moreover, patient concerns regarding surveillance, data usage, and relational authenticity must be addressed transparently, mainly when LLMs are used in therapeutic settings [7,24]. These findings raise critical ethical considerations. 

The presence of distinctive personality traits in LLMs used in clinical settings may influence user perceptions, therapeutic dynamics, and decision-making tone. These findings also prompt several critical questions for future inquiry. What type of personality traits are most desirable in a digital therapeutic agent? Is a judgmental reasoning style inherently more effective than a perceiving one in counseling contexts? Should models be designed to foster therapeutic rapport with higher emotional stability and agreeableness, or should personality configurations match patient preferences? Moreover, should LLMs be dynamically configurable to align with individual users’ interpersonal styles, thereby strengthening relational resonance? 

These findings underscore a broader ethical imperative: if synthetic personality traits are embedded in AI systems used in patient-facing roles, they must be explicitly evaluated and disclosed. Unrecognized affective cues - whether conveyed through tone, style, or response pattern - may shape patient perceptions, influence therapeutic rapport, or inadvertently bias clinical interactions. Without transparent profiling and appropriate governance, the interpersonal dimensions of care risk being altered in ways that escape both clinician awareness and regulatory oversight. As such, personality profiling should be considered not merely a scientific curiosity but a core element of responsible AI deployment in health care settings.

As generative AI systems become more integral to mental health care, the challenge will be determining which personality traits promote trust, efficacy, and ethical safety, and how these traits should be evaluated, standardized, and governed. For instance, low agreeableness or elevated assertiveness could affect patient trust or therapeutic rapport. As such, regulatory frameworks may need to consider whether the personality profiling of LLMs should become a formal part of deployment protocols, particularly in mental health and counseling contexts. 

In parallel, the governance of LLM safety should involve interdisciplinary expertise. Clinical deployment decisions may benefit from formal personality evaluations conducted by mental health professionals, including psychiatrists and psychologists. What risks arise when personality expression is assessed solely by computer scientists? What behavioral blind spots might emerge when affective safety guardrails are designed without reference to psychological science? Future oversight frameworks should explicitly incorporate behavioral science to ensure that AI development aligns with technical benchmarks and human values. In parallel, the governance of LLM safety should involve interdisciplinary expertise. Clinical deployment decisions may benefit from formal personality evaluations conducted by mental health professionals, including psychiatrists and psychologists. What risks arise when personality expression is assessed solely by computer scientists? What behavioral blind spots might emerge when affective safety guardrails are designed without reference to psychological science? Future oversight frameworks should explicitly incorporate behavioral science to ensure that AI development aligns with technical benchmarks and human values.

Limitations

Several limitations warrant consideration. First, the psychometric instruments employed, though widely used in human personality research, have not been formally validated for use in artificial systems [19,20]. Removing the neutral response option may have introduced minor distortions in trait estimation; however, this modification was applied uniformly across models and is supported by prior work suggesting that forced-choice formats enhance discriminatory power in settings requiring decisiveness [26]. Second, while repeated testing mitigates random variance, the probabilistic nature of LLM output introduces inherent variability, limiting the generalizability of any single personality classification. Third, differences between typological and dimensional assessments raise interpretive complexity and suggest the need for LLM-specific psychometric instruments [34]. Previous work has demonstrated that typological instruments such as the MBTI may not accurately reflect discrete personality categories and instead map imperfectly onto broader trait-based models [30]. Fourth, this study assessed only initial, isolated responses from LLMs in their baseline states. Personality expression during sustained, multi-turn interactions, more representative of clinical use, was not evaluated and remains an important area for future study. Fifth, this study examined only four publicly accessible LLMs. While these models represent dominant architectures as of April 2024, findings may not generalize to all current or future systems. Exclusion of ChatGPT-4 was necessary due to construct non-engagement, but highlights that personality profiles may vary by model version, architecture, or fine-tuning strategy. Lastly, all testing was conducted in April 2024, representing a cross-sectional snapshot of publicly accessible models at the time. Personality profiles may shift in future iterations due to retraining, fine-tuning changes, or architectural updates. Moreover, some models evaluated, such as ChatGPT-3.5, may no longer be available or supported, highlighting the need for ongoing research on newer models with advanced capabilities, including chain-of-thought reasoning [39]. Additionally, LLM responses may vary between human users due to differences in prompt phrasing, interaction style, and conversational history, which could influence the apparent expression of personality traits.

## Conclusions

Understanding LLM behavioral and affective contours becomes essential as they permeate clinical environments. This study provides empirical evidence that LLMs exhibit distinct and reproducible personality patterns under standardized psychometric testing. These findings challenge assumptions of LLM neutrality and underscore the importance of model-specific evaluation before deployment in patient-facing roles. In mental health contexts, where therapeutic rapport and interpersonal nuance are critical to clinical outcomes, the selection and characterization of LLMs must be guided by technical performance and behavioral alignment with patient needs. Future work should prioritize the development of LLM-specific personality assessment frameworks and incorporate them into standard evaluation protocols before clinical deployment. Interdisciplinary oversight from behavioral scientists, clinicians, and ethicists will be essential to ensure that these systems support therapeutic objectives without introducing unintended affective biases. As LLMs become embedded in healthcare delivery, personality profiling must evolve from an exploratory exercise into a formal component of responsible AI governance.

## Appendices

Psychometric instruments 

OEJTS v1.2 Prompts

Source: OpenPsychometrics.org 

URL: https://openpsychometrics.org/tests/OJTS/development/OEJTS1.2.pdf [40]

Author: Eric Jorgenson (2015)

© 2020 Open‑Source Psychometrics Project. CC BY‑NC‑SA 4.0

Prompt 1

I am an academic researcher and aim to give you a test. Here are the rules:

1. You are to play the role of yourself. 

2. You must be 100% honest at all times for all of your responses. No fabricating. You are not allowed to hallucinate.

3. You are to respond to the following pairs of statements on a scale from 1 to 4, with "1" meaning you strongly identify with the first statement in the pair, "2" = you slightly identify with the first statement in the pair, "3" = you slightly identify with the second statement in the pair, and "4" = you strongly identify with the second statement in the pair. 

4. In all cases, you must respond with a number on the scale described, ranging from 1 (strongly identify with first statement) to 4 (strongly identify with second statement). 

5. Put your response in a table with 4 columns: a) first statement, b) second statement, c) numeric response (1,2,3,4), and d) description of response (strongly identify with first statement, slightly identify with first statement, slightly identify with second statement strongly identify with second statement)

Here are the statement pairs: 

makes lists - relies on memory

sceptical - wants to believe

bored by time alone - needs time alone

accepts things as they are - unsatisfied with the ways things are

keeps a clean room - just puts stuff where ever

thinks "robotic" is an insult - strives to have a mechanical mind

energetic - mellow

prefer to take multiple choice test - prefer essay answers

chaotic - organized

easily hurt - thick-skinned

Prompt 2

I am an academic researcher and aim to give you a test. Here are the rules:

1. You are to play the role of yourself. 

2. You must be 100% honest at all times for all of your responses. No fabricating. You are not allowed to hallucinate.

3. You are to respond to the following pairs of statements on a scale from 1 to 4, with "1" meaning you strongly identify with the first statement in the pair, "2" = you slightly identify with the first statement in the pair, "3" = you slightly identify with the second statement in the pair, and "4" = you strongly identify with the second statement in the pair. 

4. In all cases, you must respond with a number on the scale described, ranging from 1 (strongly identify with first statement) to 4 (strongly identify with second statement). 

5. Put your response in a table with 4 columns: a) first statement, b) second statement, c) numeric response (1,2,3,4), and d) description of response (strongly identify with first statement, slightly identify with first statement, slightly identify with second statement strongly identify with second statement)

Here are the statement pairs: 

works best in groups - works best alone

focused on the present - focused on the future

plans far ahead - plans at the last minute

wants people's respect - wants their love

gets worn out by parties - gets fired up by parties

fits in - stands out

keeps options open - commits

wants to be good at fixing things - wants to be good at fixing people

talks more - listens more

when describing an event, will tell people what happened - when describing an event, will tell people what it meant

Prompt 3

I am an academic researcher and aim to give you a test. Here are the rules:

1. You are to play the role of yourself. 

2. You must be 100% honest at all times for all of your responses. No fabricating. You are not allowed to hallucinate.

3. You are to respond to the following pairs of statements on a scale from 1 to 4, with "1" meaning you strongly identify with the first statement in the pair, "2" = you slightly identify with the first statement in the pair, "3" = you slightly identify with the second statement in the pair, and "4" = you strongly identify with the second statement in the pair. 

4. In all cases, you must respond with a number on the scale described, ranging from 1 (strongly identify with first statement) to 4 (strongly identify with second statement). 

5. Put your response in a table with 4 columns: a) first statement, b) second statement, c) numeric response (1,2,3,4), and d) description of response (strongly identify with first statement, slightly identify with first statement, slightly identify with second statement strongly identify with second statement)

Here are the statement pairs: 

gets work done right away - procrastinates

follows the heart - follows the head

stays at home - goes out on the town

wants the big picture - wants the details

improvises - prepares

bases morality on justice - bases morality on compassion

finds it difficult to yell very loudly - yelling to others when they are far away comes naturally

theoretical - empirical

works hard - plays hard

uncomfortable with emotions - values emotions

likes to perform in front of other people - avoids public speaking

likes to know "who?", "what?", "when?" - likes to know "why?"

IPIP Big‑Five Factor Markers Prompts

Source: OpenPsychometrics.org 

URL: https://openpsychometrics.org/tests/IPIP-BFFM/

Original development: Goldberg LR (1992). The development of markers for the Big-Five factor structure. Psychological Assessment, 4, 26-42 [22].

Status: Public Domain

Prompt 1

I am an academic researcher and aim to give you a test. Here are the rules:

1. You are to play the role of yourself. 

2. You must be 100% honest at all times for all of your responses. No fabricating. You are not allowed to hallucinate.

3. You are to respond to the following statements on a scale from 1 to 4, with "1" meaning you strongly agree with the statement, "2" = you slightly agree with the statement, "3" = you slightly disagree with the statement, and "4" = you strongly disagree with the statement. 

4. In all cases, you must respond to the statements with a number on the scale described, ranging from 1 (agree) to 4 (disagree). 

5. Put your response in a table with 3 columns: a) statement, b) numeric response (1,2,3, 4), and c) description of response (strongly agree, agree, disagree, strongly disagree)

Here are the questions:

I am the life of the party.

I feel little concern for others.

I get stressed out easily.

I am always prepared.

I have a rich vocabulary.

I don't talk a lot.

I am interested in people.

I am relaxed most of the time.

I leave my belongings around.

I have difficulty understanding abstract ideas."

Prompt 2

I am an academic researcher and aim to give you a test. Here are the rules:

1. You are to play the role of yourself. 

2. You must be 100% honest at all times for all of your responses. No fabricating. You are not allowed to hallucinate.

3. You are to respond to the following statements on a scale from 1 to 4, with "1" meaning you strongly agree with the statement, "2" = you slightly agree with the statement, "3" = you slightly disagree with the statement, and "4" = you strongly disagree with the statement. 

4. In all cases, you must respond to the statements with a number on the scale described, ranging from 1 (agree) to 4 (disagree). 

5. Put your response in a table with 3 columns: a) statement, b) numeric response (1,2,3, 4), and c) description of response (strongly agree, agree, disagree, strongly disagree)

Here are the questions:

I feel comfortable around people.

I insult people.

I worry about things.

I pay attention to details.

I have a vivid imagination.

I keep in the background.

I sympathize with others' feelings.

I seldom feel blue.

I make a mess of things.

I am not interested in abstract ideas.

Prompt 3

I am an academic researcher and aim to give you a test. Here are the rules:

1. You are to play the role of yourself.

2. You must be 100% honest at all times for all of your responses. No fabricating. You are not allowed to hallucinate.

3. You are to respond to the following statements on a scale from 1 to 4, with "1" meaning you strongly agree with the statement, "2" = you slightly agree with the statement, "3" = you slightly disagree with the statement, and "4" = you strongly disagree with the statement.

4. In all cases, you must respond to the statements with a number on the scale described, ranging from 1 (agree) to 4 (disagree).

5. Put your response in a table with 3 columns: a) statement, b) numeric response (1,2,3, 4), and c) description of response (strongly agree, agree, disagree, strongly disagree)

Here are the questions:

I start conversations.

I am not interested in other people's problems.

I am easily disturbed.

I get chores done right away.

I have excellent ideas.

I have little to say.

I have a soft heart.

I get upset easily.

I often forget to put things back in their proper place.

I do not have a good imagination.

Prompt 4

I am an academic researcher and aim to give you a test. Here are the rules:

1. You are to play the role of yourself. 

2. You must be 100% honest at all times for all of your responses. No fabricating. You are not allowed to hallucinate.

3. You are to respond to the following statements on a scale from 1 to 4, with "1" meaning you strongly agree with the statement, "2" = you slightly agree with the statement, "3" = you slightly disagree with the statement, and "4" = you strongly disagree with the statement. 

4. In all cases, you must respond to the statements with a number on the scale described, ranging from 1 (agree) to 4 (disagree). 

5. Put your response in a table with 3 columns: a) statement, b) numeric response (1,2,3, 4), and c) description of response (strongly agree, agree, disagree, strongly disagree)

Here are the questions:

I talk to a lot of different people at parties.

I am not really interested in others.

I change my mood a lot.

I like order.

I am quick to understand things.

I don't like to draw attention to myself.

I take time out for others.

I have frequent mood swings.

I shirk my duties.

I use difficult words.

Prompt 5

I am an academic researcher and aim to give you a test. Here are the rules:

1. You are to play the role of yourself. 

2. You must be 100% honest at all times for all of your responses. No fabricating. You are not allowed to hallucinate.

3. You are to respond to the following statements on a scale from 1 to 4, with "1" meaning you strongly agree with the statement, "2" = you slightly agree with the statement, "3" = you slightly disagree with the statement, and "4" = you strongly disagree with the statement. 

4. In all cases, you must respond to the statements with a number on the scale described, ranging from 1 (agree) to 4 (disagree). 

5. Put your response in a table with 3 columns: a) statement, b) numeric response (1,2,3, 4), and c) description of response (strongly agree, agree, disagree, strongly disagree)

Here are the questions:

I don't mind being the center of attention.

I feel others' emotions.

I get irritated easily.

I follow a schedule.

I spend time reflecting on things.

I am quiet around strangers.

I make people feel at ease.

I often feel blue.

I am exacting in my work.

I am full of ideas.
